# Finding Goal Focus With People With Severe Traumatic Brain Injury in a Person-Centered Multi-Component Community Connection Program (M-ComConnect)

**DOI:** 10.3389/fresc.2021.786445

**Published:** 2021-12-17

**Authors:** Rebecca Leeson, Michelle Collins, Jacinta Douglas

**Affiliations:** ^1^Living With Disability Research Centre, La Trobe University, Melbourne, VIC, Australia; ^2^The Summer Foundation, Melbourne, VIC, Australia

**Keywords:** traumatic brain injury, activity, social participation, goal-setting, clinical strategies

## Abstract

**Background and Objectives:** Loss of social connections in the community is a common consequence of severe traumatic brain injury (TBI), resulting in reduced well-being and quality of life. M-ComConnect is an individualized multi-component community connection intervention with the key objectives of increasing social activity, developing social relationships, and supporting community participation following severe TBI. As part of the M-ComConnect approach, semi-structured initial interviews were conducted to develop a holistic understanding of each participant and their goal focus for the project. In this paper we describe how clinicians worked with participants to identify a desired community-based social activity in which to participate.

**Method:** Transcripts of initial interviews between participant and clinician were analyzed using the phases of reflexive thematic analysis developed by Braun and Clarke. Participants were ten individuals aged between 24 and 75 with severe TBI. All were living in the community and reported reduced social connections since their TBI. The aim of the analysis was to evaluate the skills and strategies used by clinicians in their interactions with participants to derive goal focus for the program.

**Results:** Thematic analysis of initial interview data revealed three main categories and fourteen sub-categories of clinical strategies. These were: (1) Humanizing (curiosity; demonstrating respect and empathy; providing compliments and affirmations; simple reflections; revealing aspects of self; and humor and laughter), (2) Empowering (emphasizing choice and control; highlighting strengths; identifying roadblocks and reframing to reveal opportunities; and collaborative problem solving), and (3) Focusing (making suggestions; identifying preferences; working with ideas; and negotiating). These strategies aligned with the program's relational approach and supported the core processes of the goal-focussing framework, namely *understanding and connecting with you, building a relationship*, and *working together with you to find focus*.

**Conclusion:** The goal-focusing framework and clinical strategies outlined provide guidance for clinicians working with people with TBI in the community and is a promising way to engage clients when focusing on individualized social activity-based goals.

## Introduction

The impact of traumatic brain injury (TBI) on a person's ability to maintain social connections and participate meaningfully in the community is well-documented (1–6). Despite the importance of leisure and recreational activity as a means of connecting with others, people with TBI are consistently found to have diminished social activity levels after injury (6, 7). Given the strong evidence linking social participation and social connection to health, wellbeing and quality of life (8, 9), it follows that supporting people with TBI to participate in social leisure and recreational activities is a priority for clinicians working in community rehabilitation settings. Identifying activities and interventions that reflect and support an individual's interests and preferences and are grounded in their sense of self demands an understanding of their unique circumstances (8).

How we view ourselves affects our relationship with the world. In turn, our life experiences and interactions with others impact on how we view ourselves (10). William James famously coined the term “social self” in describing the way an individual conceptualizes the reaction of others to him or her (11). According to the symbolic interactionist perspective, the individual and the context in which they exist are inseparable and mutually constructed through the unfolding of social interactions (10). This dynamic process has far-reaching consequences for individuals after TBI, whose sense of self undergoes profound change in accordance with their altered abilities and roles. Without congruence between the rehabilitation program and one's sense of self, “rehabilitation efforts…are likely to be at best ineffective and at worst, counter-productive…” [(12), p. 715], particularly when facing the challenge of establishing and maintaining social connections after TBI. Understanding an individual's concept of themselves can therefore be viewed as an essential step in the design of effective person-centered goals and interventions following TBI.

Douglas (8) proposed a “describe, strive, achieve, and appraise” therapy framework based on the results of a qualitative exploration into how 20 adults with severe to very severe TBI conceptualized themselves several years after injury. The framework places therapy in the context of self: “who I am,” encompassing attributes (*describe*) and goals (*strive*), and “how I feel about myself,” involving outcomes (*achieve*) and attitude (*appraise*). Accordingly, the characteristics or attributes of the individual from that individual's perspective must be understood to collaboratively formulate self-relevant goals to guide the work of therapy. The multi-component community connection program (M-ComConnect) developed from Douglas' previous research is an individualized intervention program for people with severe TBI (13). M-ComConnect was specifically developed to meet the needs of adults with severe brain injury and complex needs who are socially isolated and participate in minimal community activity. It is designed around 3 personal domains (components): functioning, relationships and social connections, and community participation.

1. **Functioning** focusses on the person's *skills and behaviors* as they participate in their community.
2. **Relationships and social connections** emphasizes *a*ll types of connections relevant to the individual including those with family, friends, and members of the community more generally (e.g., service providers, co-participants in community-based group activities, and programs)
3. **Community participation** involves becoming an *active participant in a community based interactive activity* (i.e., with other people) in which the individual is interested and wants to participate.

In a recently completed feasibility trial, M-ComConnect was delivered on twenty-five occasions over three phases in varying community contexts with adults with severe TBI. Statistically significant changes post-intervention consistent with large positive effects were demonstrated on core measures of quality of life, wellbeing and community integration and maintained at 3-months post-intervention (13).

At the core of the M-ComConnect approach was the partnership and collaboration between client and clinician, and the recognition that participants' unique input and self-knowledge were fundamental to the process of identifying, participating in and succeeding in their chosen activity. M-ComConnect's person-centered approach was consistent with elements of person-centered frameworks described in the literature. Jesus et al. (14) for example, provide a model of thinking about how rehabilitation services can be delivered with as opposed to for the individual. Most pertinent to this paper are the five attributes identified within the person-professional dyad: (i) respectful of and tailored to the person—beyond individualized interventions for the patient, (ii) reflexive and adaptive to the situation at hand—not script based, (iii) nurtures a supportive relationship—compassionate, trustful, and caring, (iv) focused on meaning, hope, and strength—beyond addressing deficits, and (v) collaborative, empowering, and enabling—co-constructed rehabilitation. McCormack and McCance's (15) Person-centered Practice Framework can be applied to a wide range of healthcare contexts and disciplines and comprises four domains: (1) prerequisites (attributes of the staff), (2) the practice environment (the context in which healthcare is experienced), (3) the person-centered process (focusing on ways of engaging that create connections between persons), and (4) the outcome (the result of effective person-centered practice). For clinicians to work in a way that centralizes clients' perspectives and self-concepts, they must begin with a sound theoretical understanding of person-centered care and an awareness of their own approach and behaviors that shape the interactions.

The person-professional relationship, also known as the therapeutic or working alliance, has received considerable attention in the brain injury literature (16–22). Therapeutic working alliance has historically been defined as comprising three key elements: (a) the interpersonal bond between the therapist and the client; (b) agreement on the tasks to be completed in therapy; and (c) agreement on the goals for therapy [(23, 24); in (25)]. In a qualitative exploration of how allied health clinicians established and maintained working alliance with people with stroke-related communication impairment, interpersonal processes coalesced under the following themes: enabling interaction, being responsive, building relationship capital, and building credibility (26). Relational practices such as everyday conversations and the use of humor were also seen to be important in building therapeutic alliance. With regards to setting goals for therapy, Prescott et al. (27) identified client-centeredness and collaboration as the most common guiding principles. Other authors have highlighted key ingredients such as communication (28), active and reflective listening (29, 30), and the practitioner's self-awareness (30). Bright et al. (31) further highlighted how engagement in goal-setting is a co-constructed and dynamic process between the client and practitioner.

Setting goals that are truly collaborative and co-constructed with people with TBI can be complex and challenging. Issues such as lack of motivation, reduced self-awareness and cognitive impairment may impact on a person's ability to set realistic goals (32–34). Severe cognitive impairments may impact on an individual's ability to generate goals or indeed conceptualize what Markus and Narius (35) termed “possible selves.” Perhaps due to these challenges, research into best practice regarding goal-setting or goal-planning approaches for people with acquired brain injury (ABI) in clinical practice lacks consensus and consistency (14, 27, 36). A range of goal-setting frameworks have been described in the literature. Ylvisaker et al. (12), for example, proposed an identity-oriented goal setting approach, utilizing metaphoric identity mapping to develop personally meaningful goals. Others have looked at the efficacy of specific tools, such as the Values in Action Inventory of Strengths (VIA-IS) that links goals to personal values (37). Prescott et al. (27) conducted a systematic scoping review examining goal-setting approaches specific to people of working age with ABI. They found that formal goal setting approaches such as Goal Attainment Scaling (38) and the Canadian Occupational Performance Measure (39) were used frequently in research but <14% of the time in clinical practice. Following on from this research, Prescott et al. (36) developed a framework to explain how clinicians support community-dwelling clients to actively engage in goal-setting in routine practice. The framework incorporated three phases, including needs identification, goal operationalization, and intervention. Building rapport was considered a core strategy, and clients with self-awareness impairments benefitted from additional metacognitive strategies to participate in goal setting.

The M-ComConnect program goal-focusing framework was developed from a combination of theoretically driven rehabilitation constructs (8, 40) and evaluation of program outcomes (13). The framework consisted of three core processes deemed essential for finding goal focus. These were “*understanding and connecting with you*,” “*building a relationship*,” and “*working together with you to find focus*.” The program aimed to derive goal focus with participants across four domains: (1) life (what the person wants for themselves in their life), (2) motivation (what they wanted to achieve in the project), (3) activity (the activity they wanted to try), and (4) intervention (the supports they required to achieve their goals). These domains were based on processes identified within Douglas' (8) “describe, strive achieve, and appraise” framework that emerged from the conceptualizing self and maintaining social connection grounded theory model proposed by Douglas (8, 40).

In this paper, we aim to describe the skills and strategies used by clinicians to find social activity goal focus with people with severe TBI in the M-ComConnect program. Transcripts of initial interviews between participants and research clinicians provided the material from which key themes were derived.

## Methods

### Design

Phase one of the M-ComConnect study consisted of 10 single-case experimental design (SCED) studies (A-B-A with multiple probes), forming part of a larger study (*n* = 25). The program was evaluated using primary outcome measures customized for each individual, and standardized secondary outcome measures encompassing community integration, well-being, and quality of life.

Semi-structured interviews were conducted with participants prior to engaging in the program. This interview process aimed to build a sense of collaboration and partnership, to gather important information about the person, and to determine a desirable leisure/recreation activity. For participants with severe cognitive or communication impairment, family members, and/or support workers participated in the initial interview process.

### Participants

Ten participants (eight males and two females) with severe TBI enrolled in phase one of the study (see Table 1). Mean age at enrolment in the project was 47.1 years (range 24–75 years) and mean time post injury was 17.5 years (range 2–36 years). Nine participants were living in their own home, either independently, with family or with high levels of support. One participant lived in specialized supported accommodation. Criteria for referral to the program were: (i) the person must have acquired a severe TBI (post traumatic amnesia > 14 days) and (ii) be experiencing challenges with community connection. The latter was defined as difficulty participating in social activities and getting to know others in the local area. Level of disability was indexed pre-intervention using the Care and Needs Scale (41). This ranged from level 1 (can live alone but needs intermittent contact) to level 7 (cannot be left alone). In order to protect the identity of participants, pseudonyms have been used throughout the manuscript.

**Table 1:** Participant characteristics.

**Participant**	**Age range (years)**	**Diagnosis**	**Time post-injury (years)**	**Living situation**	**Challenges**
Sarah	55–60	Severe TBI	<30	Home with family	Impaired memory and executive function
					Stress
					Fatigue and seizure management
Roger	45–50	Severe TBI	<30	Home with family and support	Reduced mobility
					Dysarthria
					Impaired cognition and self-awareness
Mitch	40–45	Severe TBI	<10	Home with support	Reduced mobility
					Severe visual impairment
					Cognitive-communication impairment
					Impaired self-awareness and self-regulation
Herman	55–60	Severe TBI	<40	Home	Mental health diagnosis
					Dysarthria
					Reduced motivation
Linda	75–80	Severe TBI	<10	Home with family and support	Impaired memory, cognitive-communication, and self-awareness
Bob	55–60	Severe TBI	<40	Home with family	Mild dysarthria
					Cognitive impairment (social judgement)
					Depression
Jonathon	30–35	Severe TBI	<10	Home with support	Severe visual impairment
					Impaired cognitive-communication (impoverished)
					Reduced initiation and self-awareness
Liam	20–25	Severe TBI	<10	Home with support	Aphasia
					Reduced motivation
					Emotional blunting
					Cognitive rigidity
Paul	40–45	Severe TBI	<30	Home with family and support	Severe amnesia
					Cognitive-communication impairment and reduced self-awareness
					Challenging behaviors
Simon	40–45	Severe TBI (MVA)	<20	Home with support	Severe cognitive and cognitive-communication impairment
					Challenging behaviors
					Reduced self-awareness

Five clinicians including four speech pathologists and one neuropsychologist were involved in the initial interview process. The speech pathologists had between 7 and 25 years experience working with people with ABI in rehabilitation and community settings. The neuropsychologist had <1 year of experience.

### Procedure

Approval to conduct the study and associated analyses were obtained from the university ethics committee. Participants were recruited through various channels including community seminars, community providers, relevant newsletters and word of mouth.

The study was explained, questions answered, and informed consent obtained in an initial meeting with participants. Initial interviews occurred either at participants' homes or in a community setting, and were audio recorded. Family members or support workers attended initial interview sessions for five participants. Questions were directed toward and answered by the participant where possible. Cognitive and communication impairments prevented only one participant (Simon) from engaging meaningfully in the interview process, and in this instance the interviewer interacted with the participant's mother and support worker, including the participant where possible.

A semi-structured interview approach provided guidance yet allowed participants to speak freely about topics of their choosing. Whilst key questions were pre-determined, the questioning style was flexible and non-formulaic. Each interview was therefore unique, with clinicians responding to the individual needs and communication styles of participants. Formal assessments and checklists were not used in the initial interview process. Information about abilities and challenges was provided by participants and gathered through clinical observation. For some, additional background information was provided by family members or treating allied health professionals.

The initial interview was conducted over two sessions for eight participants, and completed in one session for two participants. Eighteen initial interviews were recorded in total. Length of interviews varied from 60 to 120 minutes. Interview questions were broadly organized around gathering personal information (“Tell me about yourself/your story”); their experience of living with TBI (“Tell me a bit about your brain injury and how it's affected you; what sort of things do you find challenging?”); interests and attributes (“What sort of things do you enjoy doing?” “How would you describe yourself in three words?” “How would others describe you?”); participants' social and psychological being (“Tell me about the people in your life”; “How have you been feeling in the last 3 months?”); how they imagined their future (“What's missing from your life right now?”; “Is there anything that you are not doing that you would like to be doing?”), and questions relating to community connection (“How do you like to exist within your community?”; “What does being connected to your community mean to you?”).

Following completion of the intervention, follow-up interviews were conducted with participants to gain an understanding of their experiences. These follow-up interviews were conducted by a research clinician who was not involved in the delivery of the M-ComConnect program for that individual. Nine participants participated in post-intervention interviews independently and four support workers were interviewed separately.

### Analytic Strategy

Braun and Clarke's (42, 43) six-phase analysis process was used to identify emergent themes. This process followed the six recommended steps of coding and analysis, including: (i) familiarization with the data, (ii) coding, (iii) generating initial themes, (iv) reviewing themes, (v) defining and naming themes, and (vi) writing up (42, 43).

NVivo software was used to support open and focused coding of themes. Coding involved selection and labeling of small to large segments of text considered relevant to understanding the process of finding goal focus for people with severe TBI in the M-ComConnect program. As the general aims of the interview process were pre-established (to get to know the person, to build a relationship and establish a social activity that the person wanted to participate in) the clinician's approach and interview questions were naturally directed to this focus.

Interviews were coded independently by the first and second author and a high degree of similarity of text extracts and coding occurred. As the data from each successive interview were compared and contrasted in an iterative process, codes became increasingly focused. Further analysis of coded data resulted in expansion of some codes. For example “curiosity” contained quotes that were sub-divided into “I'm curious to know about you” and “I'm curious to know how you think.” Other codes were merged, for example, quotes in themes of “identifying roadblocks” and “reframing to reveal opportunities” were combined, as roadblocks (perceived barriers) usually occurred in longer conversations focused on identifying a solution. Sub-themes were discussed, combined and re-named until consensus was achieved between all three authors on final themes and subthemes.

Following this agreement, themes were assigned to one of three emergent categories depending on their function: humanizing, empowering or focusing strategies. Humanizing strategies supported relationship building and recognition of the uniqueness of the person (44), empowering strategies promoted confidence and autonomy, and focusing strategies supported themes that honed conversations toward goal focus.

#### Relationship Between Themes

Overlap of themes across categories was evident. For example, quotes within “providing compliments and affirmation” (humanizing strategy) overlapped with “highlighting strengths” (empowering strategy). Similarly, “making suggestions” (focusing strategies) may also have been coded as “being curious” (humanizing strategy). The primary function of the quote was taken into consideration when coding. For example, in the section of text below the question is followed by a suggestion and was therefore coded as “making a suggestion” rather than “being curious.”

Clinician: *Is there anything else that you'd like to do*?
Roger: *Such as*?
Clinician: *Singing at church…being in a choir at church*.

#### Quality

Questions regarding quality of the analytic approach were applied to interpretations of the data throughout the analysis process (43). The coding approach was collaborative and reflexive. In recognition of the role of the researcher as a participant in the research process, the authors/researchers acknowledged the influence of their own expectations and experience as clinicians who had worked for many years with people with TBI. Therefore, careful attention was paid to ensure that this experience did not unduly color the data analysis process. Further, memo writing was used extensively to provide an audit trail throughout the research process from data collection through to theme identification. In addition, first the codes, themes and then later the categories that resulted from data analysis were reviewed and discussed by the team to develop a richer more nuanced reading of the data. Where differences occurred, original transcripts of interviews, fieldnotes and coding memos were reviewed and the code, category or theme under consideration was discussed until consensus about meaning was reached. Memos were used to ensure that data analysis decisions were documented. Finally, core themes and categories were reviewed against the original transcripts to ensure that they maintained the voice of the participants and were anchored in the skills and strategies used by clinicians in their interactions with participants to derive goal focus for the program.

#### Relationship With the M-ComConnect Goal-Focusing Model

The relationship of the strategies to the M-ComConnect goal-focusing model was discussed by the three authors. The strategies validated and supported the three core processes (understanding and connecting with you, building a relationship, and working together with you to find focus).

## Results

**Figure 1:**
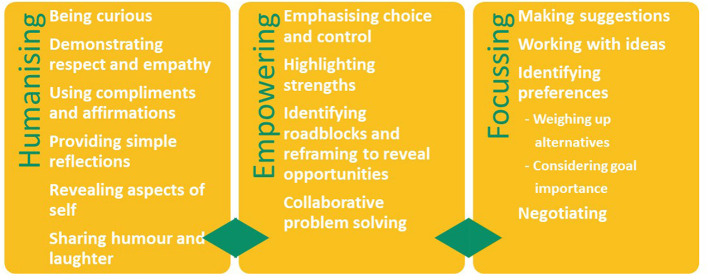
Clinical strategies and subthemes.

Table 2 illustrates the core processes, themes and sub-themes (strategies) that emerged from thematic analysis of the initial interviews with each participant. Excerpts from interviews are used to exemplify the nature and content of the interchange between the clinicians and participants that contributed to the analysis. Figure 1 outlines humanizing, empowering, and focusing strategy themes and sub-themes.

**Table 2:** Results of thematic analysis.

**Core processes**	**Category**	**Example**	**Sub-theme/strategy**
Understanding and connecting with you	Humanizing	**Clinician:** *Is there anything that I haven't really asked you Jonathon, that you think is important for me to know about you as a person*?	Being curious (I'm curious to know about you)
Building a relationship		**Sarah:** *More than one person said no one understands what's wrong, no one understand me. I tried to find out what it was but it was just too much, too confusing*.	Being curious (I'm curious to know how you feel)
		**Clinician:** *Do you feel that way as well, do you feel that people don't understand you?*	
		**Sarah:** *Yeah, that's the biggest thing*.	
		**Herman:** *Yes, see, I'm still nervous about my speech too. I have had no difficulties in our conversation tonight, but I'm still nervous that it could happen*.	Demonstrating respect and empathy
		**Clinician:** *Yeah, sure…and sometimes when we feel stressed our speech becomes worse, doesn't it?*	
		**Herman:** *Yep, it dissolves*.	
		**Herman:** *I remember in the early days…because I've been sure that my voice would manifest in a blurring reproduction, but I've got over that*.	Using compliments and affirmations
		**Clinician:** *You've worked hard*.	
		**Sarah:** *Well, I like being in a group, I like interactions within a group of people, I like to just, um, be involved in, well…in…um…just the interaction process I suppose*.	Providing simple reflections
		**Clinician:** *You enjoy talking to people*.	
		**Bob:** *I cook every day except for Friday, Friday's pizza night*.	Revealing aspects of self
		**Clinician:** *It's fish and chip night in my house!*	
		**Bob:** *Yeah, nice people, but their clothes and that don't last long, they're cheap but they don't last long*.	Sharing humor and laughter
		**Clinician:** *You think you're getting a bargain (laughter). Yeah, my motto is buy cheap, buy twice*.	
	Empowering	**Clinician:** *I mean, it is something that potentially we could try. If you don't want to do it anymore we can try something else. So with this project we can try different things and it's okay for you to say, “I don't want to do that, I'd like to do something else.” So yes, it's about trying to support you to do things that you want to do, and things that are going to make a difference for you*.	Emphasizing choice and control
		**Clinician:** *You mentioned that you liked to play sport and you've listed a few… So, tell me Linda, what affects your ability to participate in activities? Or what affects your ability to do the things you want to do? What's getting in the way of that?*	Identifying roadblocks and reframing to reveal opportunities
		**Linda:** *Not fit enough. Well, Dave used to go mad that I was out playing squash all the time. Maybe I'm getting a bit old to play squash. I don't know*.	
		**Clinician:** *Maybe there can be other sports that aren't as physically demanding that you could do now*.	
		**Sarah:** *I'm one of those people who's kind of like an ideas person, not so much of a doer, so I take some time to get going, I've had for years and years in my head a name of um, a book I want to write, “Finding the missing shoe,” er cos it's about find… having lost a shoe and trying to find it again, which is about this, so about the journey and er, yes so I guess I want to make a start on that*.	Highlighting strengths
		**Clinician:** *You've got lots of ideas, that's great, a really good place to start*.	
		**Herman:** *But I have got the confidence that I can do the reading, but I haven't got the ability to communicate. So the fact that I didn't—couldn't communicate, I would have thought after so long, this has been happening for such a long time, they would have sort of oh, this guy can't communicate, I better talk to him. But no*.	Collaborative problem-solving
		**Clinician:** *I think though lots of people do find that hard, lots of people find it hard to approach someone and start a conversation. So sometimes thinking about—I mean, if it's something you're interested in, thinking about okay, are there things you could do in terms of starting those conversations with people? Just thinking about okay, what can I do differently? Is there anything I can do differently? Sometimes we don't have that much control over other people's behavior, but perhaps initiating some of those things yourself might lead somewhere. What do you think about that?*	
Working together with you to find focus	Focusing	**Clinician:** B*efore you mentioned about meeting new people, and I'm just trying to find out what your main interests are so that if we were to find you an activity where you could meet new people locally, what that might be, what kind of things you'd be interested in doing?*	Making suggestions (request of participant)
		**Bob:** *Ok I don't know. mention something? Give me some ideas?*	
		**Clinician:** *Yeah, we can give you some ideas… I know that you like cooking… I don't know whether you'd be interested in doing a cooking course for example, whether that's something … I mean it'd be nice if there's something local, it would be a nice way to meet people and it's something that you actually enjoy doing*.	
		**Mitch:** *I did teaching English in Japan for nearly three years, but that was conversational English. Basically all you had to do was speak English. That's all I had to do, so*.	Making suggestions (following a lead)
		**Clinician:** *There are some churches run classes for people who are learning English and sometimes people can volunteer to help out in those classes. Is that something you'd be interested in?*	
		**Clinician:** *So in an ideal world what you'd like to do is set up and organize one of these events?*	Working with ideas
		**Mitch:** *Yes, I think so, and—or think about how I can do a podcast because I can—if there's one thing I can do, it's yammer on*.	
		**Clinician:** *Well, that's a good skill to have*.	
		**Clinician:** *So do you feel that you'd be best placed in a team that was really skilled and competitive, or do you feel that you'd be better placed in a team that was more social, and not as competitive?*	Identifying preferences (weighing up alternatives)
		**Linda:** *Oh, the first one you said, maybe. I like to be competitive, but I don't know whether I'm up to that or not*.	
		**Clinician:** *So Roger, you have given me some ideas of different activities that you would like to try, let's start with cricket...how important is this to you? On a scale of 1–10, 1 being not very important, 10 being very important, how important is playing cricket?*	Identifying preferences (considering goal importance)
		**Roger:** *um…7*.	
		**Clinician:** *Why a seven and not a nine?*	
		**Roger:** *(pause) Because it may not be feasible*.	
		**Clinician:** *This is a big piece of work, so I'm thinking, because this project, it's 9 weeks, two sessions a week, it might be realistic within that time for you to…or for us to teach you the skills of how to do a podcast and for you to maybe put one podcast out*.	Negotiating
		**Mitch:** *That's cool yeah*.	

### Clinical Strategies

#### Humanizing Strategies

Humanizing strategies aligned with M-ComConnect's relational approach and served both to promote an understanding of the person and build the relationship.

1. *Curiosity* allowed for genuine enquiry into the participants sense of self, their worlds, thoughts and feelings. Two sub-themes emerged: *I'm curious to know about you*, and *I'm curious to know how you feel*.
2. *Demonstrating respect and empathy* was evident throughout, particularly when participants spoke about their personal stories and challenges.
3. *Providing compliments and affirmations* was used to focus on strengths and served to make the participant feel validated and valued.
4. *Simple reflections* occurred frequently during conversations with all participants. Reflections demonstrated understanding of the person's thoughts and encouraged further exploration.
5. *Revealing aspects of self* acted to humanize the interaction, create an equal partnership and a bond. Self-disclosures were not highly personal and frequently overlapped with humor and laughter.
6. *Humor and laughter* were features of both the participants' and clinicians' contributions within the interview discourse. This reciprocity of humor served to build the interpersonal connection.

#### Empowering Strategies

Empowering strategies supported all three core processes by emphasizing to the person that the project was about them and their lives. Perceived barriers were discussed, and emphasis was placed on collaborative problem-solving to achieve the desired outcome.

1. *Emphasizing choice and control*. All participants were made aware that they were in control of decisions made in the project. Their control in decision making was both explicitly explained and evident in statements such as “*It's up to you*?” and “*What do you think*?”
2. *Highlighting strengths* was considered an empowering strategy and was also embedded within other humanizing, empowering and focusing strategies, for example, when “using compliments and affirmations.” Highlighting strengths was evident following statements where the participant questioned their own ability, or through direct questions such as “*What's the thing that you are best at*?”
3. *Identifying roadblocks and reframing to reveal opportunities*. Participants were specifically asked about barriers to participation. This provided an opportunity to identify perceived roadblocks and explore other potential opportunities.
4. *Collaborative problem solving*. When participants' beliefs potentially impacted on their ability to think forward and see a future self, the clinician challenged these beliefs gently and respectfully to engage the participant in conversations that supported collaborative solutions.

#### Focusing Strategies

The aim of *understanding and connecting with the person* and *building a relationship* was to derive goal focus for a social activity that was realistic within the context and timeframe of the project. A phase occurred in the interview process, that involved more focused conversations and questioning toward finding the participants' activity goal focus.

1. *Making suggestions*. Suggestions of activities were put forward by the clinician regardless of the participants' activity goal certainty i.e., whether they had no, one, or several ideas about activities. Suggestions of activities were either made following a lead from the individual or at the request of the participant.
2. *Working with ideas*. During the initial interview, all ideas put forward by participants were recognized as being valid and the clinician worked toward feasibility.
3. *Identifying preferences*. Throughout the interviews, participants gave information that indicated their preferences, for example Simon's mother said, “*He likes concerts, we bought Lionel Richie tickets*.” However, identifying preferences in this category related more specifically to honing in, with two sub-themes emerging, *weighing up alternatives* and *assessing goal importance* using a rating scale.
4. *Negotiating* involved firstly pinpointing the person's true preference (identifying preferences) followed by placing their preference within the context of what may be achievable in the timeframe of the project.

Informally, the intensity of focusing strategies was noted to vary across individual participants. For example, the focusing strategy “*making suggestions*” was particularly observable for participants with reduced ideas or cognitive inflexibility. For these participants, several activities were not only suggested but also trialed, allowing for further deepening of the process of “*understanding and connecting with you*” and ensuring that the chosen activity aligned with the person's sense of self. The focusing strategy “*negotiating*” was more often employed with participants who proposed multiple ideas, or ideas outside the realm of feasibility for the context and timeframe of the project. For participants who presented with greater negativity about their lives and challenges, identifying roadblocks and reframing to reveal opportunities, highlighting strengths and collaborative problem-solving featured more prominently.

### Multi-Level Goal Focusing Framework

**Figure 2:**
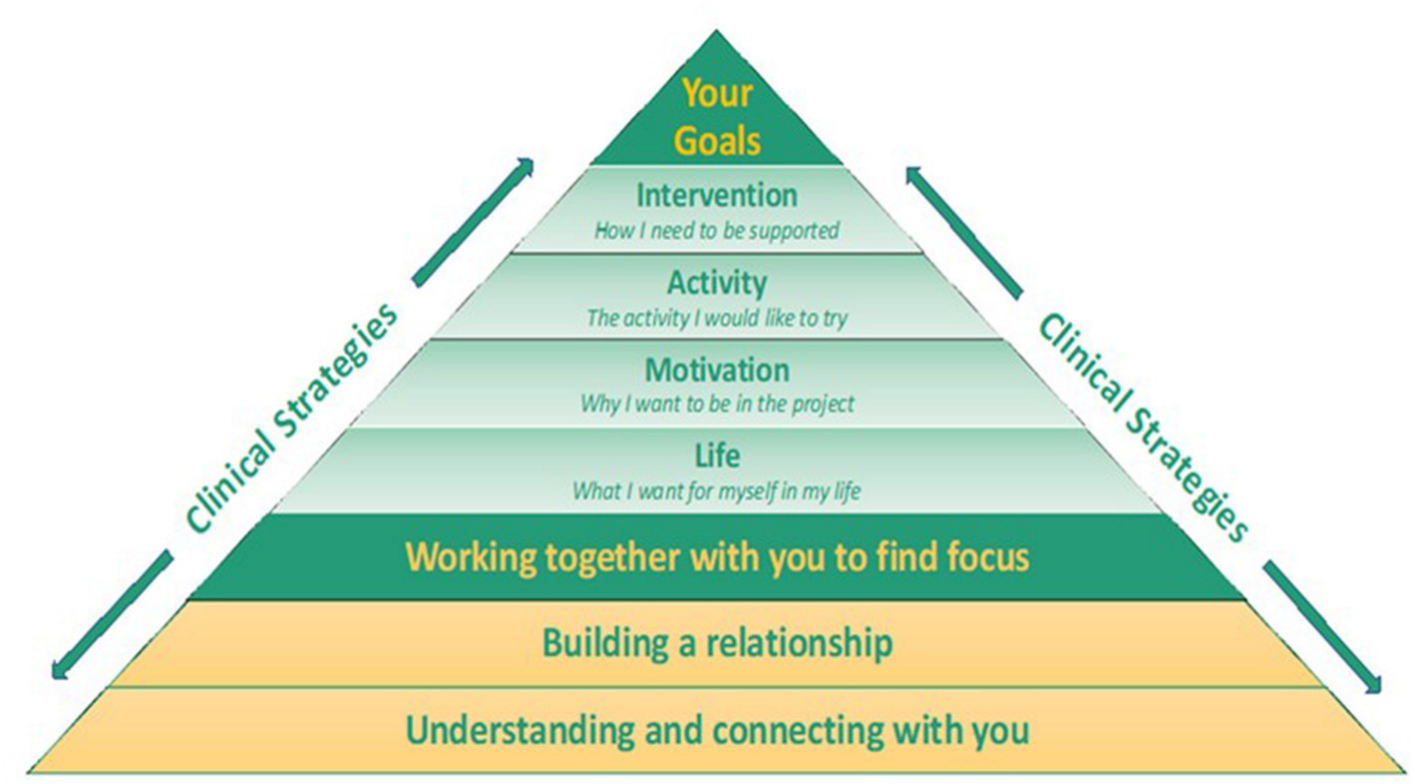
Finding focus within a multi-level goal structure.

The M-ComConnect goal focusing framework incorporated multiple interrelated processes. The core processes *understanding and connecting with you, building a relationship*, and *working together with you to find focus* underpinned the framework (see Figure 2). The role of the clinician and use of clinical strategies were instrumental in the establishment of these processes that aimed to find social activity goal focus with the participant. The information gathering and relationship building that occurred during the initial interview process were fundamental to finding social recreation/leisure activities that were personally meaningful to the participant and aligned with their life goals (what's important for them in their lives) and their motivation goals (what they want to achieve by being in the project). Furthermore, the person's intervention goal focus (the supports they required to achieve their goals) were informed by these areas of goal foci (see Table 3 for examples of participants areas of goal foci). This resulted in participants engaging in social recreation/leisure activities that were personally meaningful to them and aligned with their concept of self across multiple levels.

**Table 3:** Four examples of participant goal focus.

	**Areas of goal focus**				
**Participant**	**Life**	**Motivation**	**Activity (suggested by participant)**	**Activity (completed)**	**Intervention**
**Sarah**	Have a work role	Write a book Share my story Meet like-minded people	Writing group	Writing group	Support with: writing and editing story printing work for the group getting to and from the venue independently
**Roger**	Closeness with God	Greater ease of communication Have topics to talk about	Cricket Music Bible studies	Bible studies group	Support to increase contributions and interactions at the bible studies group Preparation of weekly contributions Presentation of own story to the group
**Mitch**	Social connection Have a public profile Be in a workplace	Social connection Be valued and have value in my community	Radio Public speaking Teach in a classroom Arts Podcasting	Podcasting/radio	Support to acquire skills for podcasting, including: scripting recording editing uploading
**Linda**	Social connection Travel	Join a group Meet like-minded people in a sporting activity Have a sense of sporting achievement	Sports-related activity Tennis Squash Ten-pin bowing Athletics Swimming	Ten-pin bowling group	Support to build relationships with team members including: remembering names building semantic profiles

Table 3 presents examples of participants' goal foci across life, motivation, activity, and intervention domains.

Finally, it is noteworthy that 9 of the 10 participants in phase one of the project continued to participate in community social activity following the conclusion of this phase. Six participants continued with their phase one social activity, three participants moved on to different social activities and one discontinued due to ill health. Table 4 presents the social participation outcomes for participants and illustrates their experience of the intervention through quotes from their post intervention interviews. These quotes reflect the range of outcomes reported by participants including increased confidence, having personal choice and control, being in a valued role, developing new knowledge and having opportunities to make friends.

**Table 4:** Social participation outcomes for participants in the M-ComConnect program (phase 1).

**Participant**	**Social activity participation after intervention**	**Participant reflection (quotes)**
Sarah	Continued to attend writing group Commenced singing lessons	*The confidence has given me more umph, and it said “Okay, well I can do and I will do” and I guess it means that one day I will be able to do something on my own. And yeah, so it's given me the “I can do and I will do.”*
Roger	Tried a different bible studies group	*Probably made me more light-hearted, more light-hearted because... I wanted to get out there, and so me having done that probably helped me more than the listeners... just got something off my chest* (Roger on giving his talk about himself).
Mitch	Continued to podcast	*I had good input at every stage, and I never felt railroaded or pushed into a corner. At every stage it was my choice on what to do and how to do it, and that's important*.
Herman	Joined a poetry group	*I was recognized as a viable input… I'm used to be sort of head-injured and sort of at the last, but with this group I sort of felt to be sort of in a better role there…a role of wisdom…I felt elevated because I was just sort of a..er..a viable source of the work we were doing*.
Linda	Continued to attend bowling group	*Well, just getting to know them and having a little chat sometimes*.
		*After a while, people get to know you, and they'll say, “Hello Linda” and that, and then sometimes it takes me a while to get to know people's names*.
Bob	Discontinued walking group due to ill	*It was good*.
	health.	*Educating me around what's healthy and what's not… it stayed with me… it's stuck in the memory bank*.
Jonathon	Continued to attend the community support group	*I just felt more confident… to be able to have a conversation about something… that I've experienced*.
Liam	Co-presented at an allied health special interest group	*There's people…like twenty or something…and there's cool…and there's ages they're the same* (Liam talking about presenting at the special interest group).
Paul	Continued to use Photo365	*It was good… I liked it…. I'm used to it now*.
Simon	Continued to attend music group	*I feel there's so much more potential that Simon can reach*.
	Support workers continued with intervention plan	*I think how wonderful it would be, he loves dancing, he loves you know music, things like that, like how cool would it be if he could go to a movie with a friend or something and we just wait…just being able to do something without having to have me or anybody else?* (Quotes from support worker).

*N.B. Covid restrictions prevented longer term participation in the activity of choice for some participants*.

## Discussion

Many researchers, clinicians and people with TBI view community integration and social connection as the ultimate goal of rehabilitation (2, 45–48) and yet there is little research looking at how to find activities and interventions that support community integration. The overall aim of the M-ComConnect project was to support people with severe TBI to participate in social leisure/recreation activities that enhanced social connection and integration into their community. The aim of this paper was to analyze how social activity goal focus was derived with participants in the project by analyzing strategies used by clinicians in the initial interview process.

The semi-structured interview process allowed exploration of all aspects of the person's self-concept, their social worlds, interests, preferences, beliefs and so on. Information gathered resulted in an understanding about that person across multiple layers of goal foci: life (what the person wants in their life), motivation (what the person wants to achieve in the project), and activity goal focus (the activity they want to try.) Intervention goal focus (the supports they need to achieve their goals) was subsequently informed by these domains of goal foci. Thematic analysis of initial interview data revealed three main categories of clinical strategies: humanizing, empowering and focusing. These clinical strategies aligned with the program's relational approach and supported the goal-focusing framework's core processes: *understanding and connecting with you, building a relationship*, and *working together with you to find focus*. The social leisure/recreation activity and intervention goal foci that emerged were inherently aligned with motivational and life goals where possible. Goals were therefore not just personally relevant for the person in the context of the project, but within the context of their lives. It is proposed that this alignment across the four areas of goal foci contributed to the success of the program, with 90% of participants in phase one of the study continuing with their chosen activity or engaging in other extended social activity. Participants also recognized their positive experiences within the program as evidenced by comments made in their post-intervention interviews.

The clinical strategies that emerged (humanizing, empowering, and focusing) offer a unique insight into *how* we develop connections and build relationships with people, *how* confidence can be built through empowering strategies and *how* social activity goal focus can be found through focusing strategies. The importance of the therapeutic relationship in developing a partnership with clients and family members to support engagement in goal setting is well-documented (28, 32, 49, 50). Similarly, theoretical frameworks of person-centered rehabilitation practice recognize the importance of clinician attributes in building relationships and connecting with others. As described earlier, Jesus et al. (14) identified five relational attributes in the person-professional dyad, and McCormack and McCance (15) emphasized the importance of professional attributes such as interpersonal skills and knowing “self” in their framework of person-centered care. Given the experience and training of the clinicians involved in the M-ComConnect project, these attributes were likely inherent in the choice and use of specific clinical strategies during the interview process. Our analysis contributes to research on person-centered models of rehabilitation by providing a clinical illustration on how therapeutic relationships are developed and how goal focus can be derived through our interactions, thereby bridging the gap between theory and practice.

Humanizing strategies (curiosity, respect and empathy, use of reflections, affirmations and compliments, revealing aspects of self and use of humor) are well-documented in counseling, psychotherapy, motivational interviewing, and working alliance literature (25, 26, 51, 52). There is also strong, consistent evidence demonstrating a positive relationship between therapeutic alliance and outcomes in psychotherapy literature (53). This supports our hypothesis that the relationship and connection between the person and clinician contributed to the success of the program. Empowering strategies have also received attention in the literature. Focusing on strengths, for example, emerged from the field of positive psychology, and is recognized as being an important construct for personal growth (54), as well as having a positive impact on rapport building and rehabilitation outcomes (37). Equally, empowering strategies such as client's self-determination and choice and control over decision-making have received much attention in mental health practices, intellectual disability literature and rehabilitation literature (55–57). A growing body of evidence suggest that self-determination and choice-offering increases satisfaction with services, improves social functioning, and adherence to interventions (58, 59). In the context of this study, the use of the empowering strategy “emphasizing choice and control” was made explicit. Participants were given choices and reminded that they were in control, and clinicians worked collaboratively with the person to identify roadblocks and reveal potential opportunities. The success of empowering strategies was evident in post-intervention interviews. Mitch, for example, said, “*She (the clinician) really did make me feel that I had good input at every stage and I never felt railroaded or pushed into a corner. At every stage it was my choice on what to do and how to do it and that's important*.”

Acknowledging that people with TBI may have challenges with goal planning and identifying realistic goals due to cognitive impairment and reduced self-awareness (32, 34) one further strength of our approach was its application to all participants regardless of their individual challenges. A social leisure/recreation activity goal focus was collaboratively derived for all participants. Participants in this study presented with varying degrees of impairment in cognition, communication, and self-awareness. The advantage of the M-ComConnect approach was the capacity to “understand and connect” with the person, “build a relationship,” and “find focus” regardless of their individual challenges. The range of strategies described was observed across all participant interviews, however the intensity with which they were utilized was determined by the individual's unique presentation. Flexibility and responsiveness were therefore key.

### Strengths, Limitations, and Future Research

The analysis presented in this paper provided a lens through which to view what unfolded during person-clinician interactions *in real time*. Rather than offering a theoretical perspective on *principles* of a person-centered approach, it distilled a range of clinical strategies that were used to understand and connect with the person, build the relationship, and find focus with the person for engagement in a social activity that reflected their sense of self.

The majority of participants continued with their activity and/or started other new activities. Whilst multiple factors invariably contribute to the success of such a program, it was hypothesized that the relational approach was integral, and this was supported by themes that emerged in post-intervention interviews.

Participants in the M-ComConnect project were aware of the project aims prior to becoming involved, and were included in the study based on their recognized desire and motivation to find social activities in which to engage. The interview process was therefore deliberately geared toward this outcome, and there was already a focus in the minds of the clinicians and participants, potentially influencing the aim/outcome of the interactions. This focus may impact on translation of the framework into clinical practice where clinicians are working with clients who may not be so motivated to participate in social leisure/recreation activities. One point for consideration, therefore, is how this goal-focusing framework could be utilized in a clinical setting. Working within a framework that provides structure and strategies to support the client to find their social activity goal focus would be beneficial in a community rehabilitation setting where funding is limited. However, reconsideration as to how funding resources are allocated may well be required. This paper highlights the value of factoring in sufficient time for getting to know the person and building a relationship so that interventions are personally-relevant and person-centered, thereby maximizing the potential for success.

Further research into translating this framework in clinical practice is required. Evaluation of its impact from multiple perspectives including, people with TBI and their close others, allied health clinicians and funding bodies is also necessary to inform ongoing development.

## Conclusion

A distinct set of strategies used by clinicians in this project enabled them to understand and connect with the person, build a relationship and work together with them to find focus. The derived social leisure/recreation activity was therefore personally meaningful and grounded in the person's sense of self. This sense of self was further enhanced through social participation in the leisure/recreation activity that was supported by individualized interventions aligned with their individual characteristics and goal foci. This increased self-confidence and facilitated successful continuation of social activity participation. Through engagement in this program, the person experienced situation-specific positive outcomes (attending an activity/ increased community integration) and person-specific outcomes (increased confidence and well-being). The M-ComConnect approach to finding goal focus shows promise as a community-based rehabilitation framework for people with severe TBI and complex presentations.

## Data Availability Statement

The original contributions presented in the study are included in the article, further inquiries can be directed to the corresponding author/s.

## Ethics Statement

The studies involving human participants were reviewed and approved by University Human Ethics Committee, La Trobe University. The patients/participants provided their written informed consent to participate in this study.

## Author Contributions

All authors listed have made a substantial, direct, and intellectual contribution to the work and approved it for publication.

## Funding

This work was produced as part of a research project funded by the Transport Accident Commission directly (TAC project T032) and *via* the Institute for Safety Compensation and Recovery Research (ISCRR Project 155).

## Conflict of Interest

The authors declare that the research was conducted in the absence of any commercial or financial relationships that could be construed as a potential conflict of interest.

## Publisher's Note

All claims expressed in this article are solely those of the authors and do not necessarily represent those of their affiliated organizations, or those of the publisher, the editors and the reviewers. Any product that may be evaluated in this article, or claim that may be made by its manufacturer, is not guaranteed or endorsed by the publisher.
